# Prevalence and Risk Factors of Eating Disorders Among Indian Adolescents

**DOI:** 10.7759/cureus.90909

**Published:** 2025-08-24

**Authors:** Hiranmayi Anam, Jagadeeswari S, Ravanagomagan MG

**Affiliations:** 1 Pediatrics, Sree Balaji Medical College and Hospital, Bharath Institute of Higher Education and Research, Chennai, IND

**Keywords:** adolescent obesity, body image dissatisfaction, eating disorders (eds), risk-factors, stress

## Abstract

Background

Eating disorders (EDs) are a growing public health concern among adolescents, yet data from India remain limited. This study examined the prevalence of EDs in adolescents aged 10-19 years in India and analyzed how age, social media use, stress, gender, socio-economic status, geographic location, and education level influence ED risk.

Methods

A cross-sectional survey of 860 adolescents in Chennai, India, was conducted using standardized questionnaires (including the Eating Attitudes Test and related scales) to screen for disordered eating and potential risk factors. Statistical analyses (independent *t*-tests, ANOVA, and linear regressions) were used to assess group differences and associations.

Results

Approximately one in ten adolescents screened positive for high ED risk, with younger adolescents (10-15 years) showing a slightly higher prevalence than older teens. Females exhibited higher ED risk scores than males, consistent with global trends. Social media use was strongly correlated with body image dissatisfaction (R≈0.82, *p*<0.001), which in turn is linked to greater ED risk. High perceived stress was a significant predictor of ED symptoms (R≈0.32, *p*<0.001). Socio-demographic factors (gender, socio-economic status, urban/rural location, education level, and family structure) all showed statistically significant variations in ED prevalence (*p*≈0.03 for each factor).

Conclusions

EDs and disordered eating behaviors are prevalent among Indian adolescents, especially in early adolescence and in females. Social media overuse and high stress levels emerge as key risk factors associated with body image dissatisfaction and ED vulnerability. These findings underscore the need for early, multifaceted intervention strategies in India, including mental health support, stress management, promotion of healthy social media habits, and culturally-tailored public health campaigns to address body image issues. Such efforts should be informed by both local data and international evidence to effectively prevent and manage adolescent eating disorders.

## Introduction

Eating disorders (EDs) are complex psychiatric conditions marked by dysfunctional eating behaviors and distorted body image. These typically emerge during adolescence and young adulthood, with global prevalence rates ranging from 5-18% in young women and 0.6-2.4% in young men [1]. While once considered issues confined to Western societies, EDs are now increasingly observed across diverse cultures. The World Health Organization (WHO) estimates that 0.5-4% of teenagers globally suffer from EDs, and mental disorders affect one in seven adolescents [2]. In India, rising awareness of adolescent EDs parallels shifts in lifestyle, diet, and media influence. However, national-level data remain sparse, and existing research often focuses on adult samples or isolated case reports. City-based surveys have estimated the prevalence of disordered eating in about 10% of Indian teens [3,4], aligning with reviews suggesting increasing ED rates in urban, affluent adolescents [4].

EDs are shaped by biological, psychological, and socio-cultural factors. Adolescence is a critical period involving hormonal changes and neurodevelopmental shifts, which can increase vulnerability to psychiatric conditions. Psychologically, risk factors include low self-esteem, perfectionism, and emotional dysregulation. Socially, media exposure and peer pressure often promote idealized body types, precipitating dissatisfaction with one’s appearance - a recognized precursor to disordered eating. In India, the collision of traditional norms with globalization and social media has introduced new body image pressures. Studies report that 10-30% of Indian adolescent girls experience body dissatisfaction [4], especially in urban areas and among high socioeconomic groups influenced by Western ideals.

Social media has emerged as a particularly relevant risk factor. Platforms like Instagram and TikTok promote appearance-centric content that encourages comparison with idealized images, increasing internalization of unrealistic body standards [5,6]. This can lead to body dissatisfaction and unhealthy eating behaviors, as documented globally. In Brazil, for example, adolescent girls with high media exposure and body dissatisfaction were significantly more likely to show ED symptoms [7]. Experimental studies also reveal that brief exposure to liked images of attractive peers can worsen body image in young women [6]. With India having one of the largest adolescent populations engaged in social media, the potential for media-driven ED risk is especially concerning.

Another important factor is psychological stress. Adolescents face pressures from academics, family, and peers, which can trigger or exacerbate eating issues. Chronic stress has been linked to disordered eating, possibly as a maladaptive coping strategy. In India, a college-based study found that students at high risk of EDs reported elevated stress and anxiety [3]. Additional risks include trauma, bullying (especially weight-related), and unstable home environments. The COVID-19 pandemic, as a global stressor, has been associated with spikes in ED cases internationally, highlighting the stress-ED connection [8].

Gender and other demographic factors also shape ED vulnerability. Globally, adolescent girls have about twice the risk of boys for developing EDs, driven by greater social pressures to conform to thin-ideal standards [1]. Though EDs also affect boys and gender-diverse youth, they often remain underdiagnosed. Socioeconomic status (SES) affects risk in multiple ways: high-SES groups may be more exposed to Western beauty ideals, while low-SES groups may experience different stressors, including food insecurity. Geographic location also matters - urban populations generally show higher ED rates, likely due to greater media exposure and changing food environments, although rural cases are now increasingly reported [4,9]. Finally, family structure and education level influence support systems and stress levels. Adolescents from single-parent homes or those with limited supervision may face increased psychosocial strain, contributing to disordered eating behaviors [4].

In summary, adolescent EDs in India likely result from a complex interplay of developmental, psychological, and sociocultural forces. This study aimed to estimate the prevalence of EDs among Indian adolescents aged 10-19 years and to investigate key risk factors including age, gender, SES, family context, stress, and social media use.

## Materials and methods

Study setting and period

This cross-sectional analytical study was carried out in the city of Chennai, Tamil Nadu, India, between July 2023 and January 2025. Chennai, being a metropolitan city with diverse socio-economic strata and a large adolescent population, served as an appropriate site to explore the prevalence and correlates of ED. The inclusion of both government and private schools and junior colleges allowed the researchers to obtain data from a representative adolescent population across various geographic zones and income groups. The timing of the study was planned to avoid exam periods, ensuring maximum participation and minimizing academic stress-induced bias.

Study population

The study population consisted of adolescents aged 10 to 19 years enrolled in selected schools and junior colleges in Chennai. Adolescence is a developmental phase marked by significant biological, psychological, and social transitions, which increase vulnerability to mental health conditions such as eating disorders. Both male and female students were included, along with a small number of non-binary individuals. Participants were recruited from grades corresponding to primary, secondary and high school levels. This population was targeted due to the heightened risk of developing body image disturbances and disordered eating behaviors during these formative years.

Sample size

The required sample size was calculated using Dobson's formula for estimating proportions in prevalence studies, based on a previously reported prevalence rate of 13% from an adolescent ED study in South India [10]. Assuming a 95% confidence level (Z = 1.96), a precision level of 3%, and accounting for a non-response rate of 10%, the final sample size was estimated at 860 adolescents. This sample size was considered sufficient to detect meaningful differences in ED risk across demographic and psychosocial variables.

Inclusion and exclusion criteria

Inclusion criteria comprised adolescents aged 10 to 19 years who were currently enrolled in the selected schools or colleges and provided informed assent, along with parental consent where applicable. Adolescents with cognitive impairments or intellectual disabilities that could interfere with questionnaire comprehension were excluded from the study. Those with diagnosed organic medical conditions, such as diabetes or thyroid disorders, were also excluded to avoid confounding factors related to body weight or appetite regulation.

Ethical considerations

The study was approved by the Institutional Human Ethics Committee of Sree Balaji Medical College and Hospital, Chennai (Reference No: 002/sBMCH/IHEC/2023/1992). Written informed consent was obtained from participants above 18 years and an assent form below 18 years from their parents or legal guardians prior to data collection. Confidentiality of responses was strictly maintained through anonymised coding of survey data. Participants were assured that their involvement was entirely voluntary, and they could withdraw at any point without any repercussions. The study adhered to national and international ethical guidelines for research involving minors.

Sampling methodology

A two-stage stratified random sampling strategy was adopted to achieve a total sample size of 860 students with equal representation across institution type and level. In the first stage, schools and junior colleges in the selected area near the medical college in Chennai were stratified into four groups: government schools, private schools, government junior colleges, and private junior colleges. From each stratum, five institutions were randomly selected from official lists, ensuring that only those providing formal permission were included. In the second stage, students were randomly chosen within each institution using computer-generated sequences to prevent selection bias. To maintain balance, 215 students were sampled from each stratum, resulting in equal representation of government and private institutions (430 students each) as well as schools and junior colleges (430 students each). On average, 43 students were selected per institution; in cases where an institution had fewer than 43 eligible students, all were included, and the shortfall was adjusted by sampling additional students from other institutions within the same stratum.

Data collection procedure

Trained research assistants visited each selected institution to conduct data collection. Participants were assembled in small groups in classrooms or auditoriums, and the survey was administered in a standardized manner. Questionnaires were provided in both English and Tamil, and students were allowed to choose their preferred language. Researchers explained the study objectives and instructions clearly and were present throughout to clarify any doubts. Anthropometric measurements, including height and weight, were taken using calibrated stadiometers and digital weighing scales, in standing position and no footwear. These measurements were used to compute the Body Mass Index (BMI) for each participant.

Operational definitions

Urban areas were defined as those falling within city corporation or municipal limits, characterized by high population density, better infrastructure, and wider access to educational, health, and commercial services. Rural areas referred to villages or panchayat-administered regions with lower population density, agriculture-based livelihoods, and relatively limited infrastructure. Suburban areas were defined as transitional zones located on the periphery of cities or towns, often under municipalities or town panchayats, displaying mixed rural-urban features. Educational institutions were categorized into primary, secondary, and higher secondary schools. Primary schools included classes 1 to 5, typically catering to children aged 6-10 years. Secondary schools comprised classes 6 to 10, usually for students aged 11-15 years. Higher secondary schools (junior colleges) included classes 11 and 12, enrolling students aged 16-18 years and serving as preparatory levels for higher education.

Study instruments

Data were collected using a structured, self-administered questionnaire comprising four main sections. The first section captured demographic and background information, including age, gender, grade level, socio-economic status (using a modified BG Prasad scale) [11], family structure (nuclear, joint, or single-parent), and area of residence (urban, suburban, rural). The second section consisted of the Eating Attitudes Test-26 (EAT-26), a validated instrument that screens for disordered eating behaviours and attitudes. A total score above 20 on the EAT-26 was used as the cutoff to identify individuals at risk for an ED [12]. The third section included the Perceived Stress Scale (PSS-10), which assessed the frequency of stress-related experiences over the past month [13]. The fourth section used the Body Shape Questionnaire (BSQ-8C) to evaluate body dissatisfaction, a well-established correlate of ED risk [14]. Permission to use the BSQ-8C was formally obtained from the original authors for the purpose of this study. Each of these tools had demonstrated reliability in previous adolescent populations and was translated and back-translated into Tamil to ensure linguistic and conceptual equivalence.

Statistical analysis

Data were entered into Microsoft Excel (Microsoft Corp., Redmond, WA, USA) and analyzed using SPSS version 26 (IBM Corp., Armonk, NY, USA). Descriptive statistics such as frequencies, means, and standard deviations were used to summarize demographic variables and scores on the EAT-26, PSS, and BSQ-8C. The prevalence of high ED risk (EAT-26 > 20) was calculated, and subgroup comparisons were performed using chi-square tests for categorical variables. All statistical tests were two-tailed, and a p-value less than 0.05 was considered statistically significant.

## Results

Based on the data collected from 860 adolescents aged 10 to 19 years in Chennai, the gender distribution in the study population was fairly balanced. There were 303 males, accounting for 35.2% of the sample, 274 females (31.9%), and 283 individuals identifying as non-binary (32.9%). Regarding age distribution, 438 participants (50.9%) were in the early adolescent age group (10-15 years), while 422 (49.1%) were in the late adolescent group (16-19 years). Socioeconomic status, categorized using the Modified BG Prasad Scale revealed that 314 adolescents (36.5%) belonged to the lower SES group (Class V), 276 (32.1%) were in the middle SES category (Class II, III & IV), and 270 (31.4%) were in the upper SES group (Class I). The geographic distribution of participants was similarly diverse, with 297 (34.5%) residing in urban areas, 286 (33.3%) from rural areas, and 277 (32.2%) from suburban regions. In terms of education level, 279 students (32.4%) were in primary school, 283 (32.9%) were attending secondary school, and 298 (34.7%) were enrolled in higher secondary school. Family structure data indicated that 303 participants (35.2%) lived in single-parent households, 271 (31.5%) in two-parent households, and 286 (33.3%) were under the care of other guardians. This diverse sample offers a representative view of adolescents across key demographic, socioeconomic, and familial strata (Table 1).

**Table 1:** Sociodemographic characteristics of study participants (n=860) * Modified BG Prasad Classification 2025 [11].

Characteristics		Frequency	Percentage
Gender	Male	303	35.2
	Female	274	31.9
	Non-binary	283	32.9
Age	10-15	438	50.9
	16-19	422	49.1
Socio-Economic Status*	Upper (Class I)	314	36.5
	Middle (Class II, III & IV)	276	32.1
	Lower (Class V)	270	31.4
Geographic Location	Urban	297	34.5
	Rural	286	33.3
	Suburban	277	32.2
Education Level	Primary School	279	32.4
	Secondary School	283	32.9
	Higher Secondary School	298	34.7
Family Structure	Single-parent household	303	35.2
	Two-parent household	271	31.5
	Guardian	286	33.3

In this study of 860 adolescents, the overall prevalence of eating disorder (ED) risk, based on EAT-26 scores ≥20, was found to be 24.7% (n=212), while 75.3% (n=648) showed no ED risk. When stratified by age, 21.0% of participants aged 10-15 years (n=92/438) and 28.4% of those aged 16-19 years (n=120/422) were at risk for EDs, indicating a higher prevalence in older adolescents. Regarding gender, 30.1% of females (n=124/412) and 19.6% of males (n=88/448) were identified as being at risk, suggesting a notably higher ED risk among adolescent girls compared to boys (Table 2).

**Table 2:** Prevalence of eating disorder risk among the study participants (n=860)

Category	n	ED Risk – n (%)	95% CI
Overall	860	212 (24.7%)	21.9% – 27.7%
Age Group			
10–15 years	438	92 (21.0%)	17.2% – 25.3%
16–19 years	422	120 (28.4%)	24.2% – 32.9%
Gender			
Male	448	88 (19.6%)	16.0% – 23.6%
Female	412	124 (30.1%)	25.7% – 34.8%

Among the 860 adolescents surveyed, several disordered eating behaviors were reported with varying prevalence and association with high eating disorder (ED) risk. A total of 146 participants (17.0%) reported experiencing eating binges, of whom 79 (54.1%) were at high ED risk. Self-induced vomiting was reported by 153 adolescents (17.8%), with 144 (94.1%) falling into the high-risk group. The use of diet pills or laxatives was noted in 135 participants (15.7%), of whom 101 (74.8%) had elevated ED risk. Excessive exercise for weight control was reported by 143 adolescents (16.6%), with 127 (88.8%) classified as high-risk. Additionally, 160 respondents (18.6%) reported losing more than 10 kg in the past six months, and 98 of them (61.3%) had high ED risk. These findings highlight the strong association between specific maladaptive weight-control behaviors and elevated risk for eating disorders in this adolescent population (Table 3).

**Table 3:** Behavioral Symptoms and Their Association with High Eating Disorder (ED) Risk (n = 860)

Behavioral Symptom	Participants Reporting Symptom, n (%)	High ED Risk Among Symptomatic, n (%)
History of eating binges	146 (17.0%)	79 (54.1%)
History of self-induced vomiting	153 (17.8%)	144 (94.1%)
History of diet pill/laxative use	135 (15.7%)	101 (74.8%)
History of excessive exercise to lose weight	143 (16.6%)	127 (88.8%)
History of weight loss >10 kg in the past 6 months	160 (18.6%)	98 (61.3%)

Among the 860 adolescents assessed, perceived stress was significantly associated with eating disorder (ED) risk. High stress was reported by 108 participants (12.6%), of whom 85.2% had high ED risk. In comparison, 17.3% of those with moderate stress (626 participants, 72.8%) and only 9.7% of those with low stress (124 participants, 14.4%) were at high ED risk (χ² = 205.3, p < 0.001). Body image dissatisfaction also showed a strong association with ED risk. Among 57 participants (6.6%) with severe concern, 86% had high ED risk; 75.6% of those with moderate concern (n = 41, 4.8%) and 23.9% with mild concern (n = 176, 20.5%) were at risk, while only 15.4% of the 586 adolescents (68.1%) with no body image concern showed high ED risk (χ² = 192.7, p < 0.001) (Table 4).

**Table 4:** Association of Stress and Body Image Dissatisfaction with High Eating Disorder (ED) Risk (n = 860) p-value <0.05 significant

Variable	Category	Total, n (%)	High ED Risk, n (%)	Chi-square (χ²)	p-value
Perceived Stress	High Stress	108 (12.6%)	92 (85.2%)	205.3	<0.001
	Moderate Stress	626 (72.8%)	108 (17.3%)		
	Low Stress	124 (14.4%)	12 (9.7%)		
Body Image Dissatisfaction	Severe Concern	57 (6.6%)	49 (86.0%)	192.7	<0.001
	Moderate Concern	41 (4.8%)	31 (75.6%)		
	Mild Concern	176 (20.5%)	42 (23.9%)		
	No Concern	586 (68.1%)	90 (15.4%)		

## Discussion

Using validated tools such as the EAT-26, PSS, and BSQ-8C, we evaluated the associations between disordered eating behavior and various psychosocial and behavioral variables in a sample of 860 participants. The findings provide important insights into the patterns of ED risk in rural, urban and suburban Indian adolescent populations, with implications for early detection and prevention. The findings of this study shed light on the significant associations between behavioral, psychological, and perceptual factors with ED risk among adolescents.

Behavioral symptoms such as eating binges, self-induced vomiting, laxative or diet pill use, excessive exercise, and recent significant weight loss were also strongly associated with ED risk in our sample. Self-induced vomiting was reported by 17.8% of participants, of whom an alarming 94.1% were classified as high risk on the EAT-26. Similarly, 88.8% of those reporting excessive exercise to lose weight were also at high risk. These findings indicate that compensatory behaviours - often under-recognised in routine adolescent health assessments - are critical warning signs of disordered eating. Prior studies have highlighted these behaviors as core components of anorexia nervosa and bulimia nervosa, where purging, restriction, or over-exercising are employed to control body weight or image [15,16].

Furthermore, the prevalence of diet pill or laxative use (15.7%) and binge eating (17.0%) in this adolescent population is concerning. Among those who reported binge eating, over half (54.1%) screened positive for high ED risk. The link between binge-purge cycles and emotional dysregulation has been well-documented, particularly in adolescence, when impulsivity and affective instability may contribute to maladaptive coping mechanisms [17]. Similarly, a weight loss of more than 10 kg in six months, reported by 18.6% of participants, was associated with high ED risk in 61.3% of those individuals. Such significant, unintentional weight changes - especially in adolescents - should prompt clinicians to evaluate for potential disordered eating behaviors.

Similarly, body image dissatisfaction (BID) was found to be a critical predictor of ED risk. Adolescents with severe BID had an ED risk of 86.0%, followed closely by those with moderate concern (75.6%). Even among those with mild concern, nearly one in four (23.9%) were at high ED risk. This corroborates earlier studies emphasizing the role of negative body image as a potent driver of eating pathology, particularly during adolescence when self-perception is highly vulnerable to external influences like media, peer comparison, and cultural ideals [18,19]. These findings are consistent with Indian studies as well, which report a growing prevalence of body dissatisfaction, especially among rural, suburban and urban adolescents exposed to Western beauty standards [20].

These findings emphasize the multifactorial nature of ED risk in adolescents, involving complex interactions between psychological stress, body dissatisfaction, and unhealthy behaviors. They also underscore the importance of screening tools like the EAT-26 and BSQ-8C in early identification of at-risk individuals. Given that these behaviors and perceptual issues often manifest subclinically before a formal ED diagnosis is made, early detection is essential. The strong statistical associations found in this study validate the importance of targeted mental health screening in school settings, where such issues often go unnoticed until they progress. Of particular note is the strong association between perceived stress levels and ED risk. Stress can influence appetite regulation, body image perception, and the use of food as a coping mechanism, all of which are implicated in ED development [21].

Limitations of the study must be acknowledged. First, this research was limited to a cross-sectional design within a single metropolitan city, which may affect the generalizability of findings to rural populations or other regions of India. Second, the use of self-report questionnaires, while standardized and validated, may introduce reporting bias. Additionally, while the EAT-26 is a widely accepted screening tool, it does not replace a clinical diagnosis from a qualified psychiatrist or psychologist. Hence, the study estimates high-risk prevalence rather than actual ED diagnoses.

Implications for practice are substantial. The data advocate for school-based awareness and intervention programs focused on stress management, healthy body image promotion, and early behavioral detection. Health professionals, educators, and parents must be sensitized to the subtle signs of ED risk beyond weight-centric measures. Given the high association of ED risk with mental health factors like stress and BID, integrative counseling programs that address emotional well-being may be beneficial. Furthermore, routine implementation of screening tools such as EAT-26 and BSQ-8C in schools could facilitate early referral and treatment, potentially preventing the development of full-syndrome eating disorders.

## Conclusions

This study highlights a significant prevalence of eating disorder (ED) risk among Indian adolescents, with nearly one in four participants (24.7%) screening positive on the EAT-26. High stress levels, body image dissatisfaction, and specific maladaptive behaviors - such as self-induced vomiting, excessive exercise, and diet pill use - were strongly associated with increased ED risk. Contrary to conventional findings, the prevalence was nearly equal among males and females, indicating a need for gender-inclusive screening and awareness strategies. These findings underscore the importance of early identification and intervention using validated tools within school and community settings. Addressing modifiable psychosocial factors such as stress and body dissatisfaction through structured mental health support and educational programs may help prevent the progression to clinically diagnosable eating disorders.
